# Supplementary material for: Lattice engineering enables definition of molecular features allowing for potent small-molecule inhibition of HIV-1 entry
Source: Nat Commun. 2019 Jan 3;10:47. doi: 10.1038/s41467-018-07851-1 (PMC6318274; doi:10.1038/s41467-018-07851-1)
Supplement: Supplementary file 1 — Supplementary Information [file 41467_2018_7851_MOESM1_ESM.pdf]

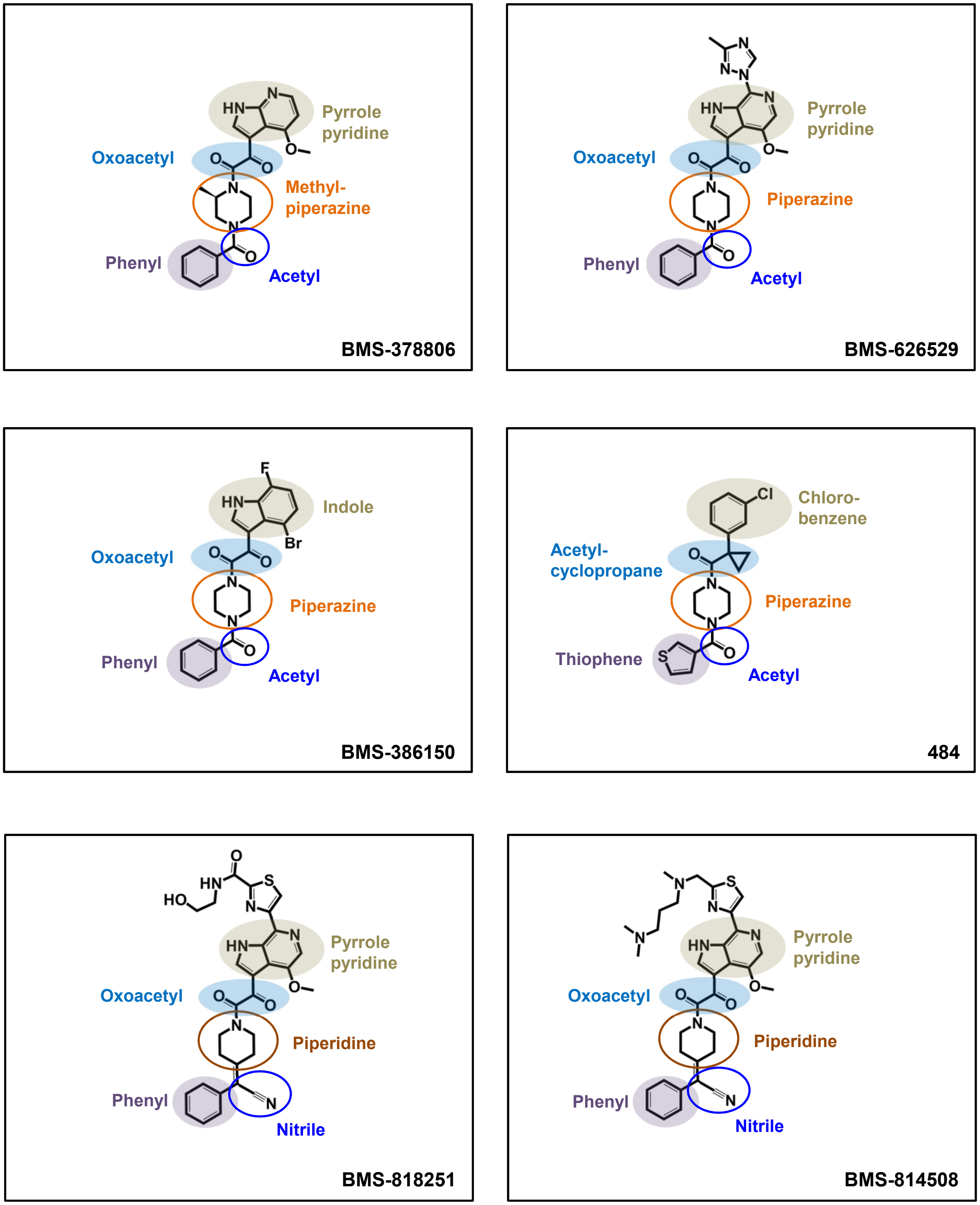

Supplementary Figure 1 | Definition of functional groups of the compounds used in this study.

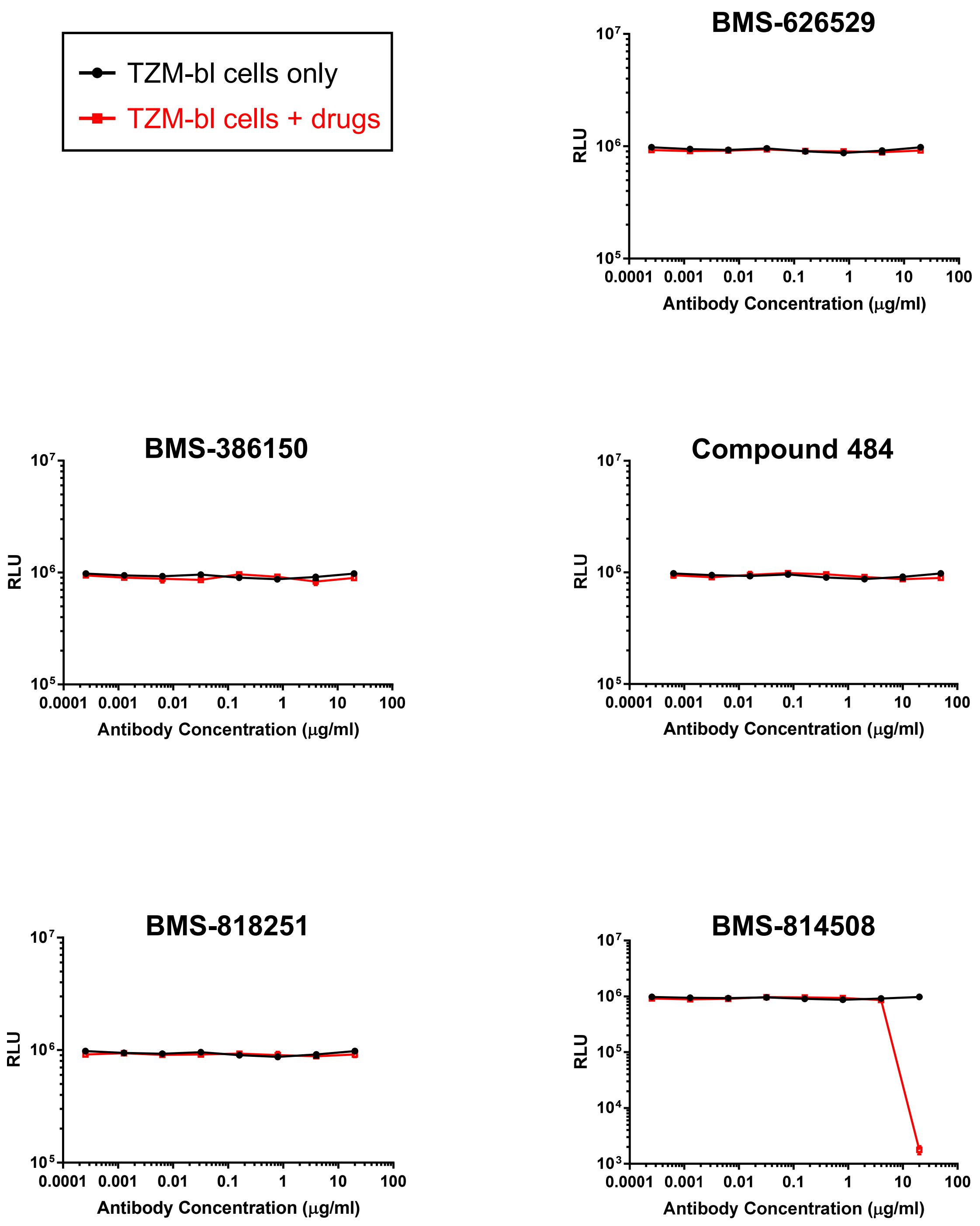

Supplementary Figure 2 | BMS-814508 showed cytotoxicity at 20uM. The lack of cytotoxicity of BMS-378806 was well studied in the literature and was not measured here.

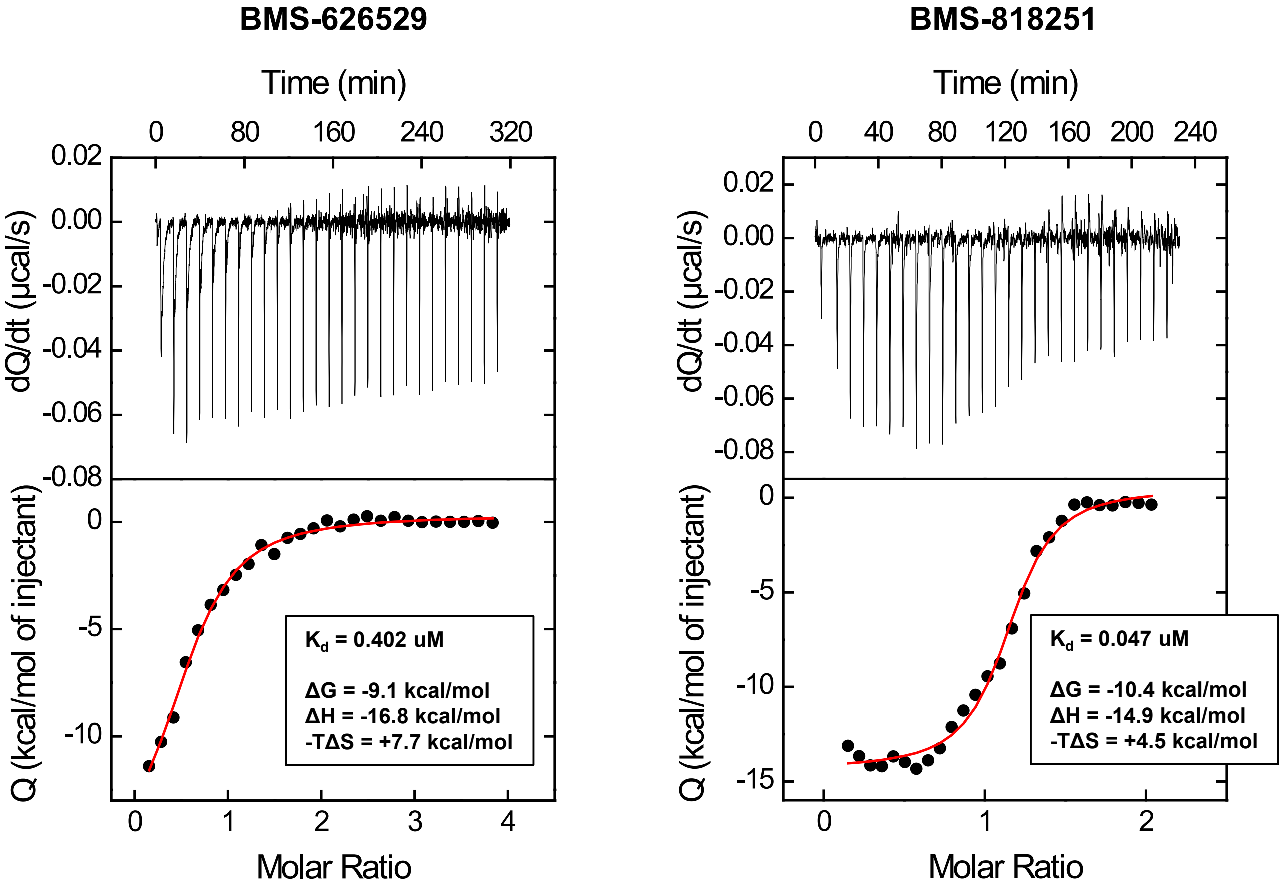

Supplementary Figure 3 | Binding affinity of BMS-818251 to BG505 DS-SOSIP, in comparison with BMS-626529 binding to BG505 DS-SOSIP, measured by isothermal calorimetry at 37ºC in the presence of 2% DMSO.

a

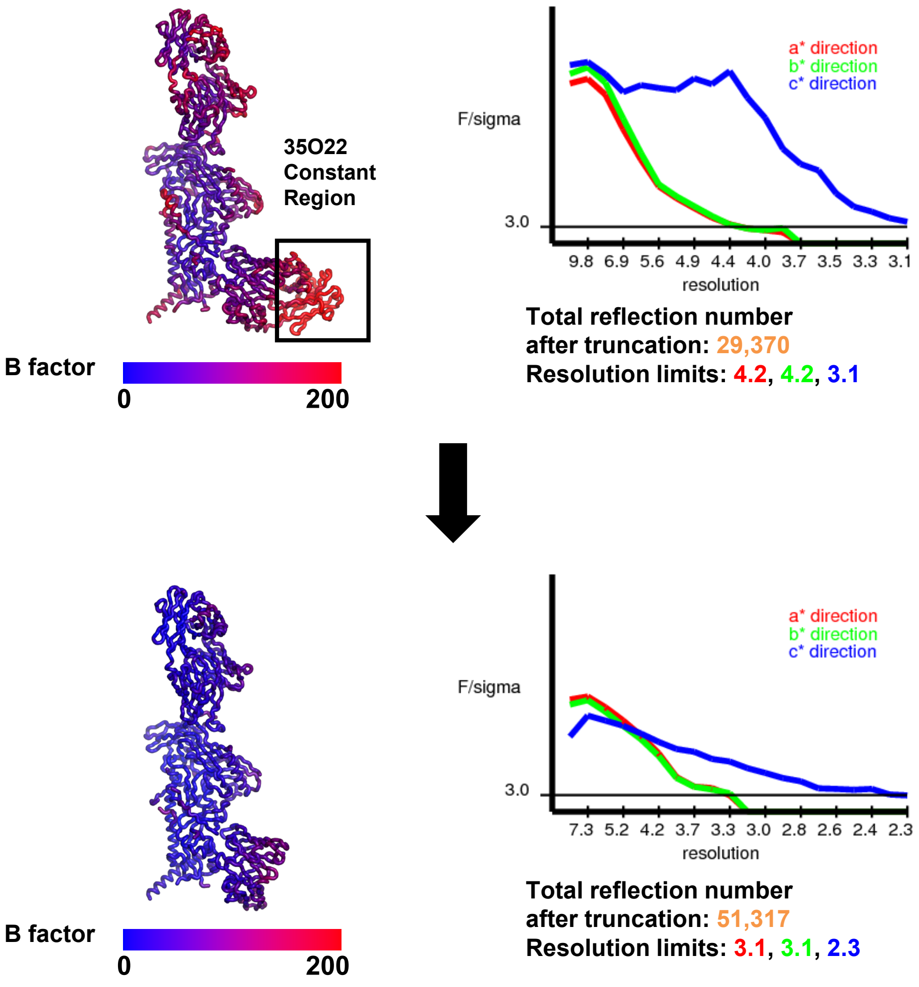

b

Generation	35O22	3H+109L	Rationale
1	scFv	-	Flexible region of 35O22 removed
2	scFv + 3T	MM	Stabilization mutations
3	scFv + 3T + 2S	MM	Further stabilization
4	scFv + 3T + 2S + 2 glycan	MM	Improved yield

Supplementary Figure 4 | Engineering of improved lattice. a, Analysis of B factors and anisotropic diffraction by UCLA anisotropy server, before and after the improvement, of the BMS-378806 complex crystal structures. **b**, Constructs of four iterations of lattice-based chaperone engineering. The two glycans that improved the production yield were introduced at ^{68}NMT^{70} (with I68N mutation) and ^{82B}NLT^{83} (with K83T mutation).

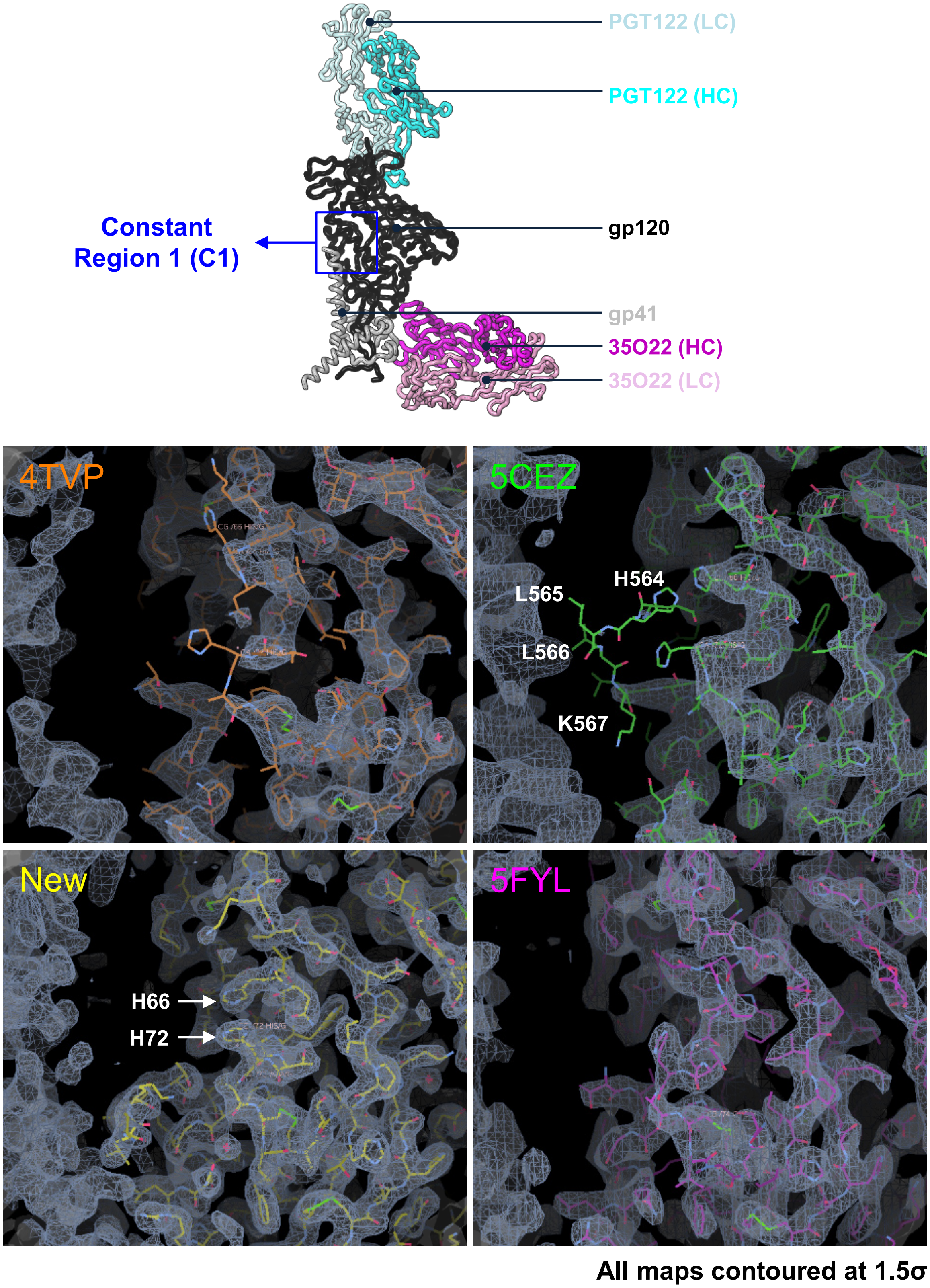

Supplementary Figure 5 | Comparison of representative density of BG505.SOSIP.664 structures determined in the P6$_3$ lattice, highlighting the C1 (constant region 1). PDB code for published structures are labeled at the top left corner of each panel. 2Fo-Fc maps are contoured at 1.5σ.

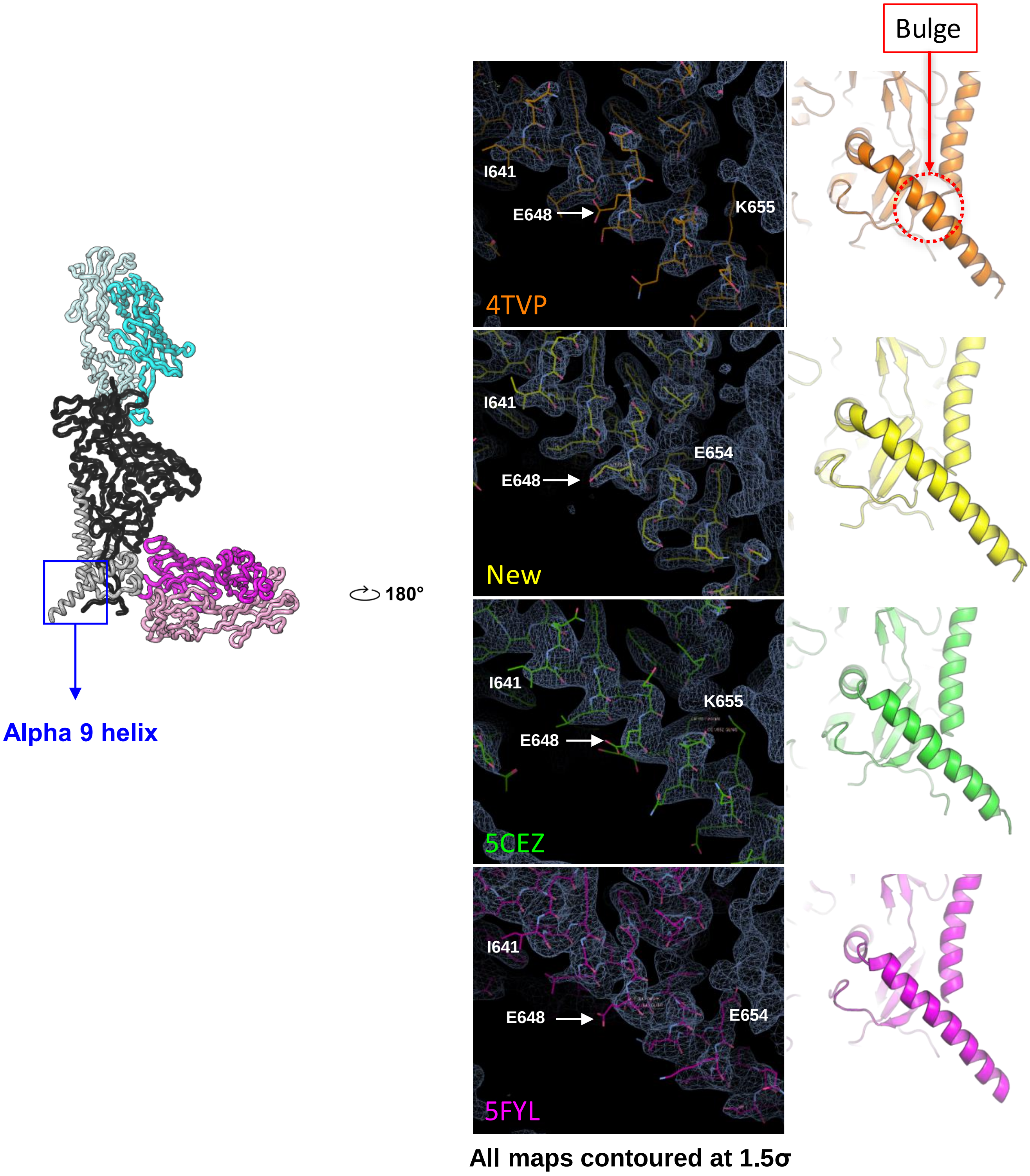

Supplementary Figure 6 | Comparison of representative density of BG505.SOSIP.664 structures determined in the P6$_3$ lattice, highlighting the alpha-9 helix. 2Fo-Fc maps are contoured at 1.5σ.

a

	Clade	BMS-814508	BMS-818251	Fold improvement
0330.v4.c3	A	0.096	0.004	24.0
BG505.W6M.C2	A	0.008	<0.0003	>26.7
KER2008.12	A	0.165	0.011	15.0
MB201.A1	A	0.192	0.014	13.7
BJOX028000.10.3	AE	>4	0.733	>5.5
C4118.09	AE	2	0.089	22.5
CNE56	AE	>4	0.471	>8.5
R3265.c6	AE	1.06	0.032	33.1
242-14	AG	0.015	<0.0003	>50.0
DJ263.8	AG	<0.0003	<0.0003	1.0
T253-11	AG	<0.0003	<0.0003	1.0
T280-5	AG	0.0008	<0.0003	>2.7
7165.18	B	0.491	0.013	37.8
B41	B	0.001	<0.0003	>3.3
BL01.DG	B	2.81	0.533	5.3
CNE10	B	0.004	<0.0003	>13.3
PVO.04	B	0.002	<0.0003	>6.7
REJO.67	B	2.73	0.45	6.1
CH119.10	BC	0.004	<0.0003	>13.3
CNE20	BC	0.143	0.006	23.8
426c	C	0.036	0.0004	90.0
CAP210.E8	C	0.4	0.022	18.2
CAP256.206.C9	C	<0.0003	<0.0003	1.0
DU156.12	C	0.02	<0.0003	>66.7
ZM106.9	C	0.031	0.0005	62.0
247-23	D	0.016	<0.0003	>53.3
A03349M1.vrc4a	D	0.008	<0.0003	>26.7
NKU3006.ec1	D	0.001	<0.0003	>3.3
X1193.c1	G	0.006	<0.0003	>20.0
P0402.c2.11	G	0.012	<0.0003	>40.0

IC50 uM
<0.001
.001-.01
.01-.100
.100-1.00
1.00-10.0
>10.0

b

	Clade	429/432	Fold change
0330.v4.c3	A	R/Q	24
BG505.W6M.C2	A	R/Q	>26.7
KER2008.12	A	R/Q	15
MB201.A1	A	R/Q	13.7
CNE56	AE	R/Q	>8.5
242-14	AG	R/Q	>50
T280-5	AG	R/Q	>2.7
X1193.c1	G	R/Q	>20
P0402.c2.11	G	R/Q	>40

	Clade	429/432	Fold change
7165.18	B	G/K	37.8
B41	B	G/K	>3.3
DU156.12	C	G/R	>66.7
247-23	D	G/R	>53.3
A03349M1.vrc4a	D	G/K	>26.7

	Clade	429/432	Fold change
CNE10	B	E/R	>13.3
PVO.04	B	E/K	>6.7
CH119.10	BC	E/R	>13.3
CNE20	BC	E/R	23.8
426c	C	E/K	90**
CAP210.E8	C	E/R	18.2
ZM106.9	C	E/R	62
NKU3006.ec1	D	E/K	>3.3

	Clade	429/432	Fold change
BL01.DG	B	K/K	5.3
REJO.67	B	R/K	6.1

	Clade	429/432	Fold change
BJOX028000.10.3	AE	G/Q	>5.5
C4118.09	AE	G/Q	22.5
R3265.c6	AE	G/Q	33.1

Supplementary Figure 7 | Fold improvement of neutralization potency between BMS-818251 and BMS-814508.

```
AENLWVTVYYGVPVWKDAETTLFCASDAKAYETEKHNVWATHACVPTDPNPQEIHLENVT
A   WVTVYYGVPVWK+A TTLFCASDAKAY+TE HNVWATHACVPTDPNPQEI L NVT
AAKKWVTVYYGVPVWKEATTTLFCASDAKAYDTEVHNVWATHACVPTDPNPQEIVLGNVT

EEFNMWKNNMVEQMHTDIISLWDQSLKPCVKLTPLCVTLQCTNVTNA---------ITDD
E FNMWKNNMVEQMH DIISLWDQSLKPCVKLTPLCVTL C NV            I+D
ENFNMWKNNMVEQMHEDIISLWDQSLKPCVKLTPLCVTLNCNNVNTNNTNNSTNATISDW

MR---GELKNCSFNMTTELRDKKQKVYSLFYRLDVVQINENQGNRSNNSNKEYRLINCNT
 +   GE+KNCSFN+TT +RDK +K Y+LFY+LDVV + EN+ N +N +   YRLINCNT
EKMETGEMKNCSFNVTTSIRDKIKKEYALFYKLDVVPL-ENKNNINNTNITNYRLINCNT

SAITQACPKVSFEPIPIHYCAPAGFAILKCKDKKFNGTGPCPSVSTVQCTHGIKPVVSTQ
S ITQACPKVSFEPIPIHYCAPAGFAILKC  K FNG+GPC +VSTVQCTHGI+PVVSTQ
SVITQACPKVSFEPIPIHYCAPAGFAILKCNSKTFNGSGPCTNVSTVQCTHGIRPVVSTQ
                                                       255

LLLNGSLAEEEVMIRSENITNNAKNILVQFNTPVQINCTRPNNNTRKSIRIGPGQAFYAT
LLLNGSLAEEE++IRSENIT+NAK I+VQ N  V+INCTRPNNNTRKSI IGPG+AFYAT
LLLNGSLAEEEIVIRSENITDNAKTIIVQLNEAVEINCTRPNNNTRKSIHIGPGRAFYAT

                                                 370
GDIIGDIRQAHCNVSKATWNETLGKVVKQLRKHFGNNTIIRFANSSGGDIEVTTHSFNCG
GDIIG+IRQAHCN+SKA WNETLG++V +L + F N TII F +SSGGD E+ THSFNCG
GDIIGNIRQAHCNISKARWNETLGQIVAKLEEQFPNKTII-FNHSSGGDPEIVTHSFNCG

                                                    434
GEFFYCNTSGLFNSTWISNTSVQGSNSTGSNDSITLPCRIKQIINMWQRIGQAMYAPPIQ
GEFFYCNT+ LFNSTW  N +      TG   +ITL CRIKQIINMWQ +G+AMYAPPI+
GEFFYCNTTPLFNSTW--NNTRTDDYPTGGEQNITLQCRIKQIINMWQGVGKAMYAPPIR
                                          424      433

GVIRCVSNITGLILTRDGGSTNSTTETFRPGGGDMRDNWRSELYKYKVVKIEPLGVAPTR
G IRC SNITGL+LTRDGG   + TETFRPGGG+MRDNWRSELYKYKVVKIEPLG+APT
GQIRCSSNITGLLLTRDGGRDQNGTETFRPGGGNMRDNWRSELYKYKVVKIEPLGIAPTA

CKRRVVGRRRRRRAVGIGAVFLGFLGAAGSTMGAASMTLTVQARNLLSGIVQQQSNLLRA
CKRRVV RRRRRRAVG+GA  LGFLGAAGSTMGAASM LTVQAR LLSGIVQQQ+NLLRA
CKRRVVQRRRRRRAVGLGAFILGFLGAAGSTMGAASMALTVQARLLLSGIVQQQNNLLRA

PEAQQHLLKLTVWGIKQLQARVLAVERYLRDQQLLGIWGCSGKLICCTNVPWNSSWSNRN
PEAQQH+L+LTVWGIKQLQARVLAVERYLRDQQLLGIWGCSGK+ICCTNVPWN SWSN+
PEAQQHMLQLTVWGIKQLQARVLAVERYLRDQQLLGIWGCSGKIICCTNVPWNDSWSNKT

LSEIWDNMTWLQWDKEISNYTQIIYGLLEESQNQQEKNEQDLLALD
++EIWDNMTW+QW+KEI NYTQ IY LLE SQ QQEKNEQ+LL LD
INEIWDNMTWMQWEKEIDNYTQHIYTLLEVSQIQQEKNEQELLELD
```

Top: BG505
Bottom: B41

Supplementary Figure 8 | Sequence alignment between BG505 and B41 Envs. Three major inhibitor contacting residues were highlighted (as defined in Figure 3 of main text) with residues highlighted in box corresponding to residues highlighted in box in Fig. 5c.

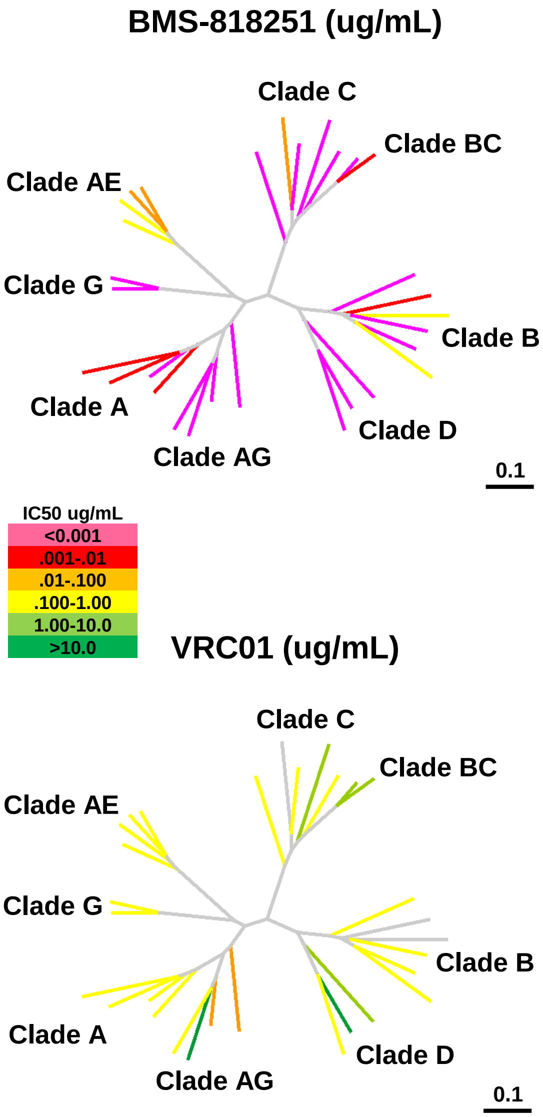

	Clade	BMS-818251	VRC01
0330.v4.c3.SG3	A	0.0023	0.1200
BG505.W6M.C2.SG3	A	<0.0002	0.1090
KER2008.12.SG3	A	0.0063	0.9510
MB201.A1.SG3	A	0.0080	0.4070
BJOX028000.10.3.SG3	AE	0.4178	0.3710
C4118.09.SG3	AE	0.0507	0.1850
CNE56.SG3	AE	0.2685	0.5970
R3265.c6.SG3	AE	0.0182	0.5760
242-14.SG3	AG	<0.0002	48.5000
DJ263.8.SG3	AG	<0.0002	0.0940
T253-11.SG3	AG	<0.0002	0.4460
T280-5.SG3	AG	<0.0002	0.0460
7165.18.SG3	B	0.0074	>50
B41.SG3	B	<0.0002	0.2290
BL01.DG.SG3	B	0.3038	>50
CNE10.SG3	B	<0.0002	0.8040
PVO.04.SG3	B	<0.0002	0.6500
REJO.67.SG3	B	0.2565	0.1450
CH119.10.SG3	BC	<0.0002	1.5900
CNE20.SG3	BC	0.0034	2.7500
426c.SG3	C	<0.0002	1.2000
CAP210.E8.SG3	C	0.0125	>50
CAP256.206.C9.SG3	C	<0.0002	0.7660
DU156.12.SG3	C	<0.0002	0.1660
ZM106.9.SG3	C	0.0003	0.8020
247-23.SG3	D	<0.0002	1.9500
A03349M1.vrc4a.SG3	D	<0.0002	12.1000
NKU3006.ec1.SG3	D	<0.0002	0.7160
X1193.c1.SG3	G	<0.0002	0.2150
P0402.c2.11.SG3	G	<0.0002	0.3900
geomean		<0.0002	0.785

Supplementary Figure 9 | Neutralization dendrogram. Phylogenetic tree showing genetic diversity of HIV-1 with branches colored according to their neutralization by BMS-818251 (top) and antibody VRC01 (bottom). Panel at right provides IC_{50} values per stains (note the unit is ug/mL, different than the uM unit used in Fig. 1b). For reference, the molecular weight of VRC01 is ~150 kDa with two active sites and the molecular weight of BMS-818251 is 570.6 Da. When compared to VRC01 (geometric mean IC_{50} 0.785 ug/mL), BMS-818251 (geometric mean ~0.0002 ug/mL) was ~30-fold more potent than VRC01 on a per-kDa, per active site basis.

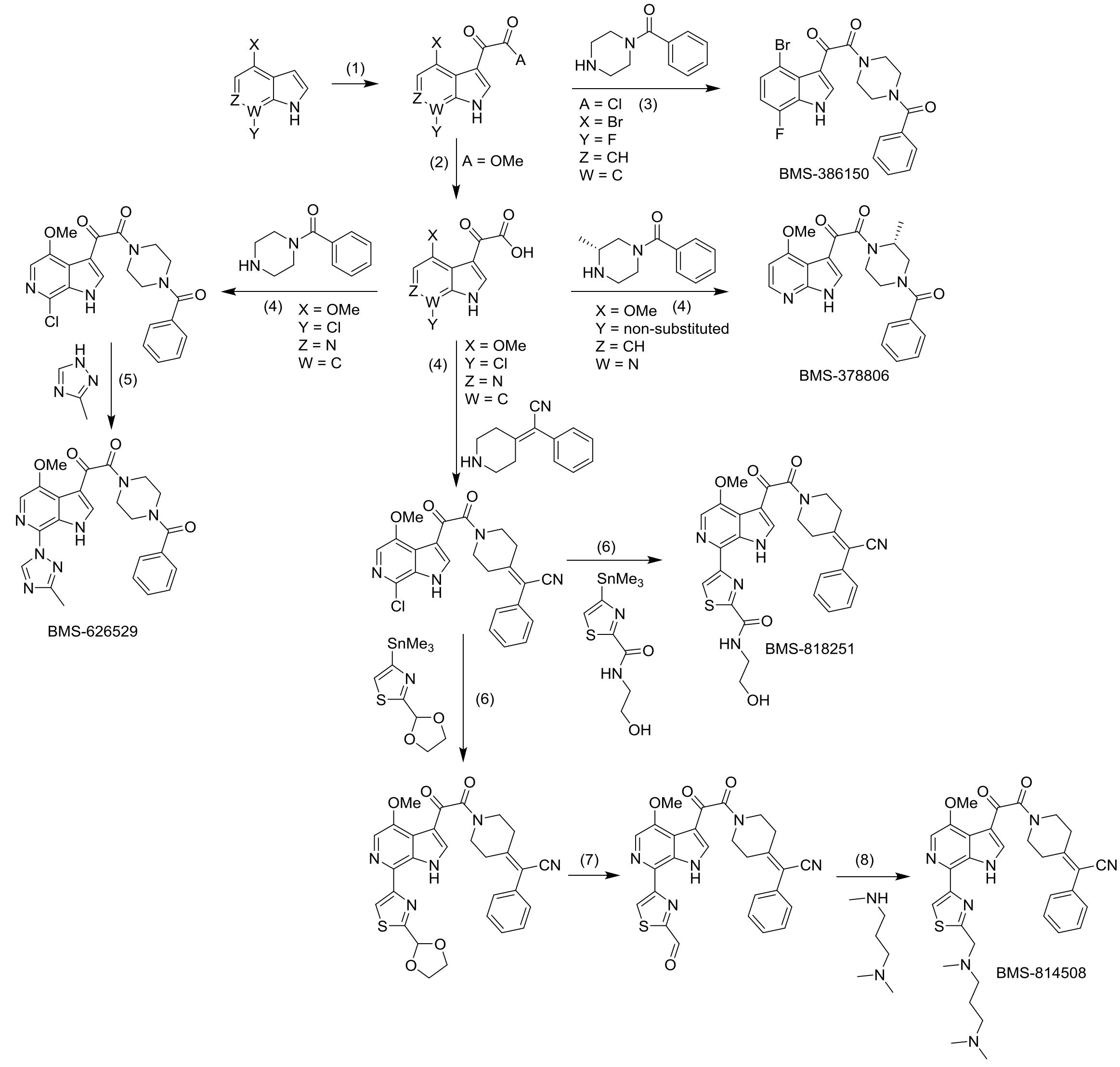

Supplementary Figure 10 | Reagents and conditions. (1) ClCOCOCl/Et2O for indole or ClCOCOOMe /AlCl3/CH2Cl2 for azaindole; (2) K2CO3/MeOH; (3) 1-benzoylpiperazine/iPr2NEt/THF; (4) 1-benzoylpiperazine or 2-phenyl-2-(piperidin-4-ylidene)acetonitrile /DEPBT/iPr2NEt/DMF; (5) 3-methyl-1H-1,2,4-triazole/Cu(0)/K2CO3/160OC; (6) 2-(1,3-dioxolan-2-yl)-4-(trimethylstannyl)thiazole or N-(2-hydroxyethyl)-4-(trimethylstannyl)thiazole-2-carboxamide/Pd(PPh3)4/dioxane/100OC; (7) TFA/H2O/70OC; (8) N1,N1,N3-trimethylpropane-1,3-diamine/NaBH(OAc)3/HOAc.

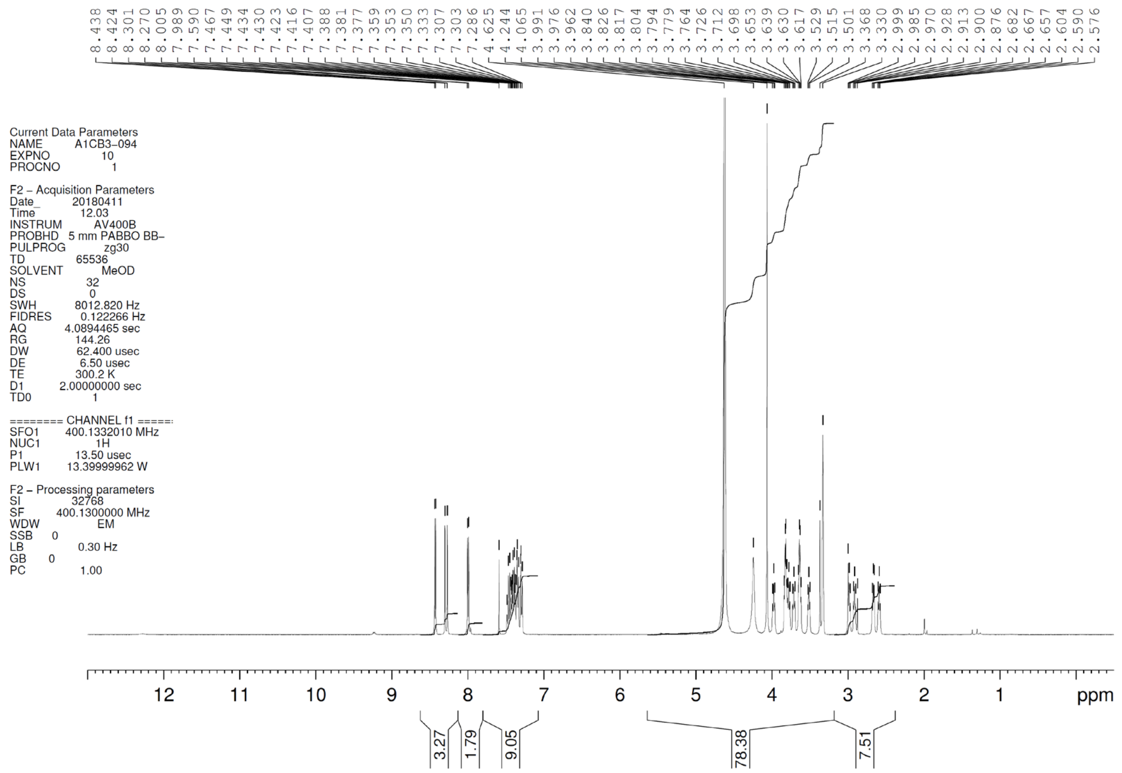

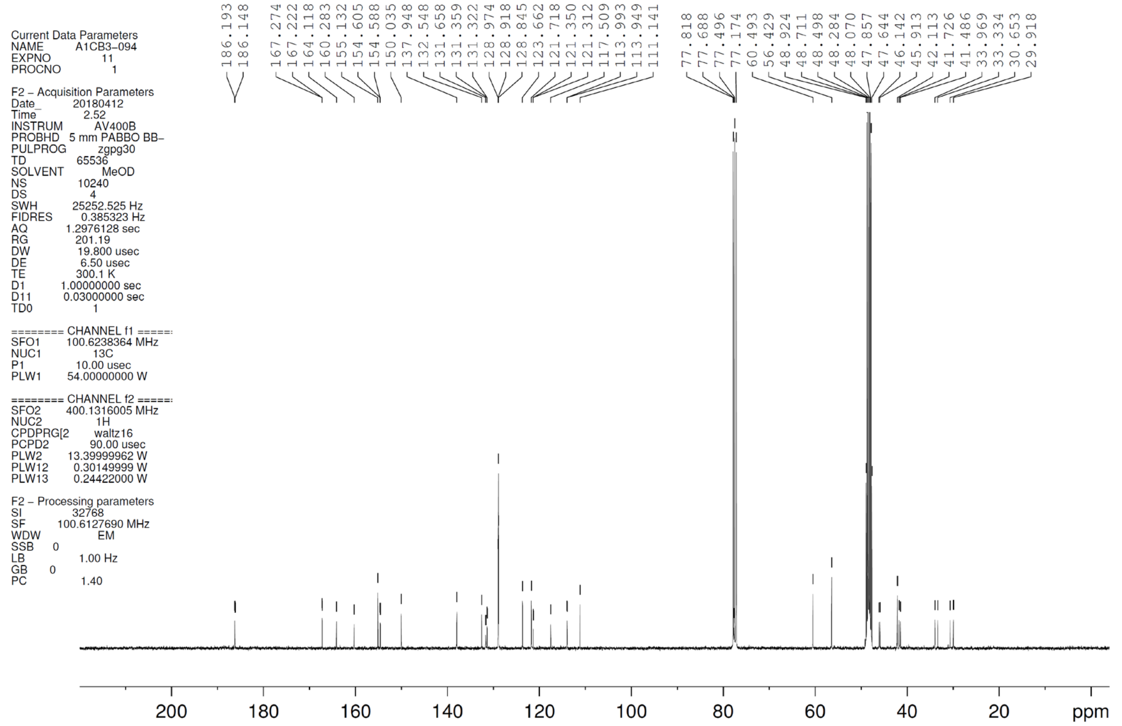

Supplementary Figure 11 | Characterization of BMS-818251 by NMR and mass spectrometry. 1H (top) and 13C (bottom) NMR spectra and high-resolution mass spectrum (next page).

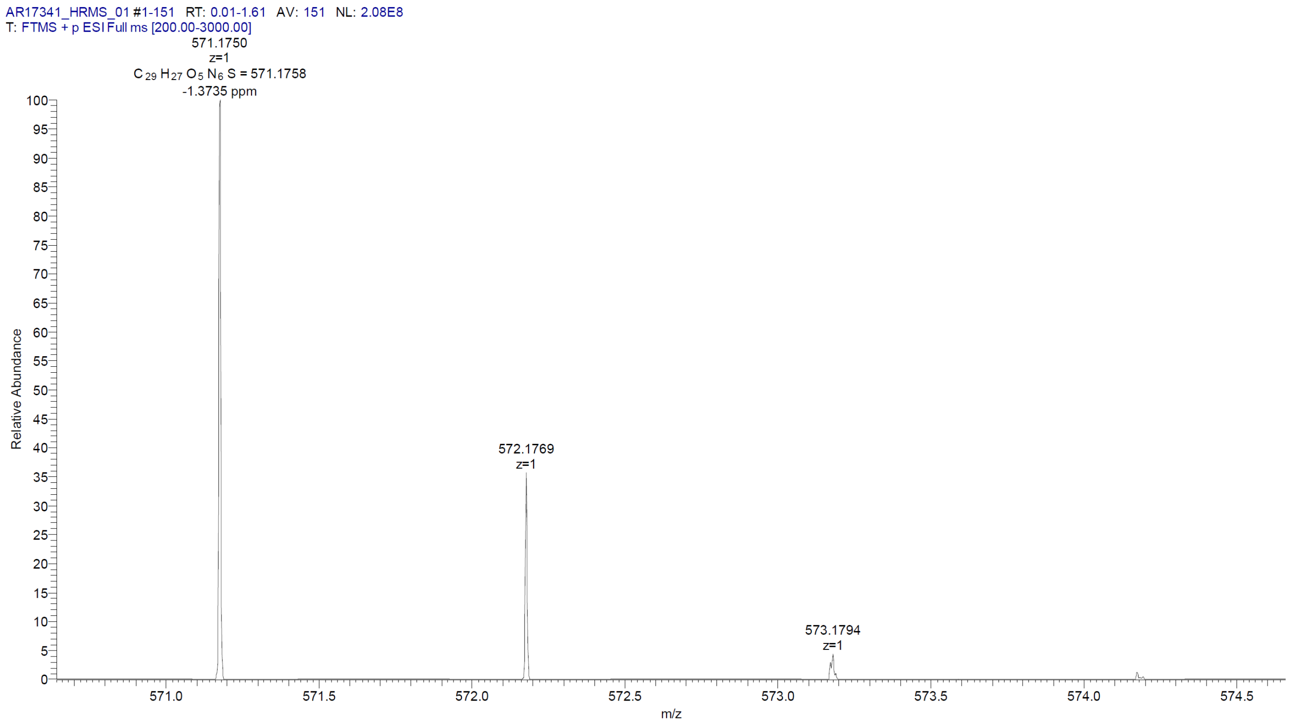

Supplementary Figure 11 (Continued) | Characterization of BMS-818251 by NMR and mass spectrometry. 1H and 13C NMR spectra (previous page) and high-resolution mass spectrum.

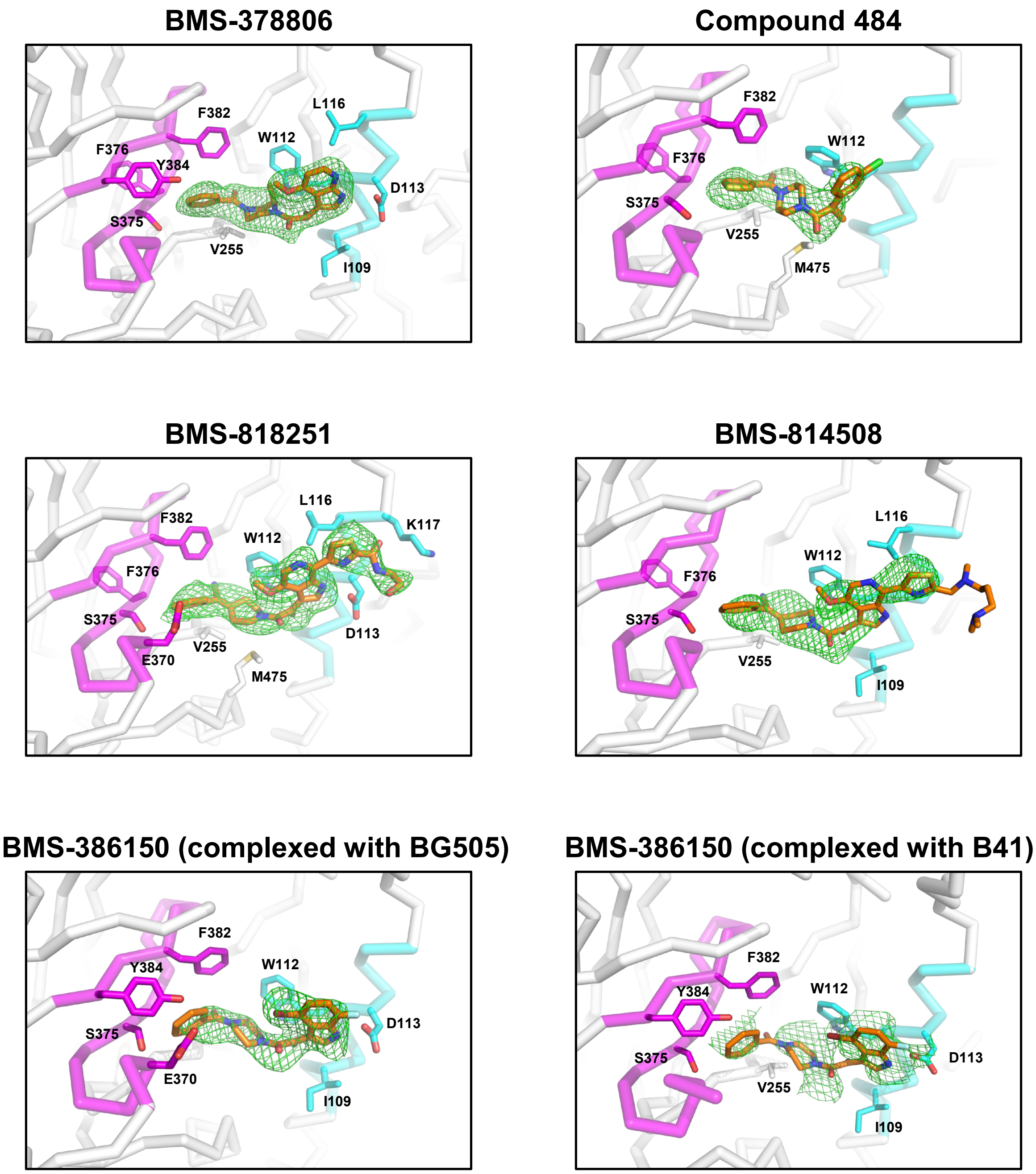

Supplementary Figure 12 | Simulated annealing difference omit map for inhibitors in the complex structures. Fo-Fc maps (contoured at 3σ) surrounding inhibitors were generated after removing inhibitors in the model and subjected the Phenix refinement with simulated annealing protocol to remove model bias.

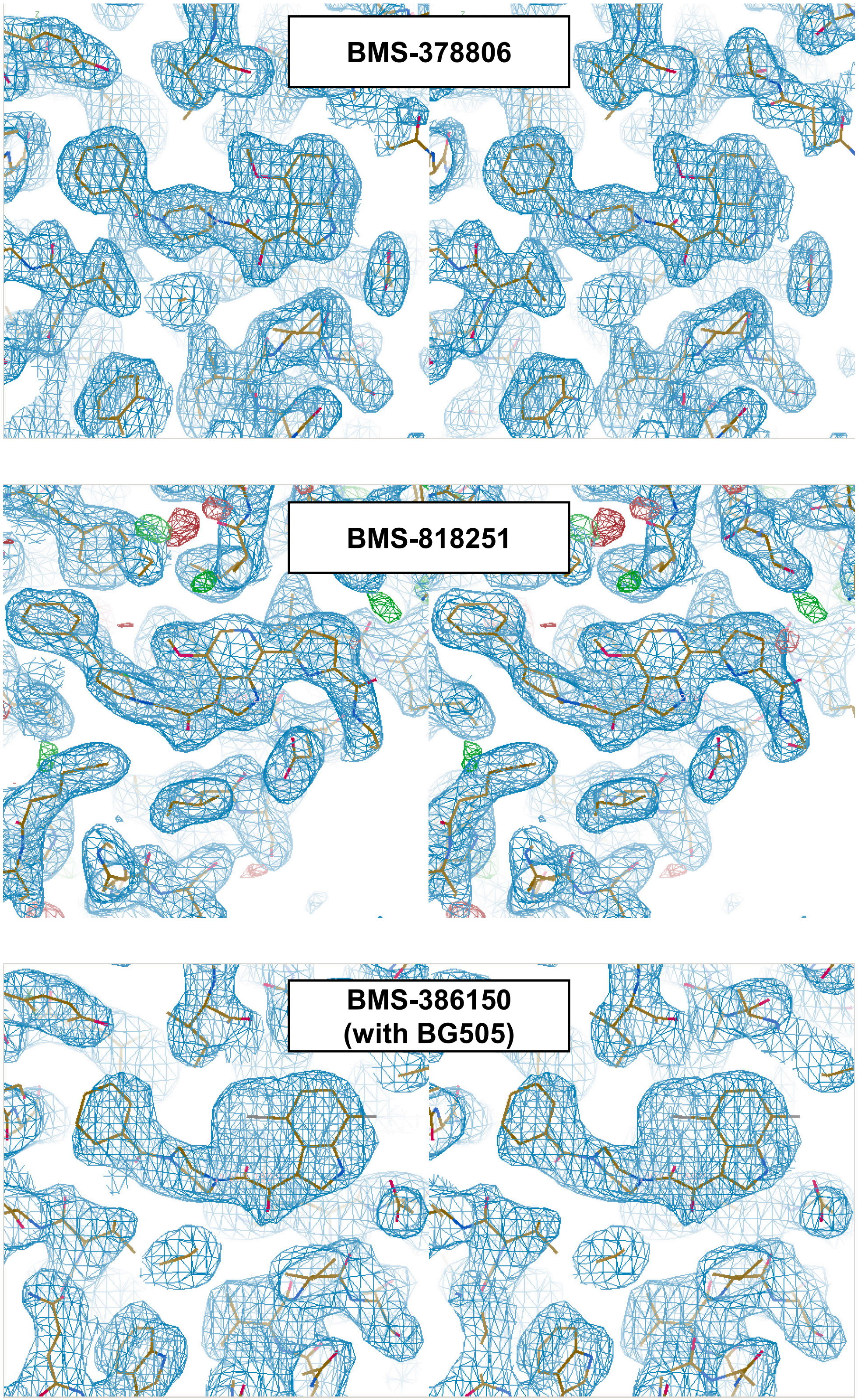

Supplementary Figure 13 | Stereo images of the entry inhibitor binding site for various drug binding. 2Fo-Fc maps, contoured at 1.5σ, are shown.

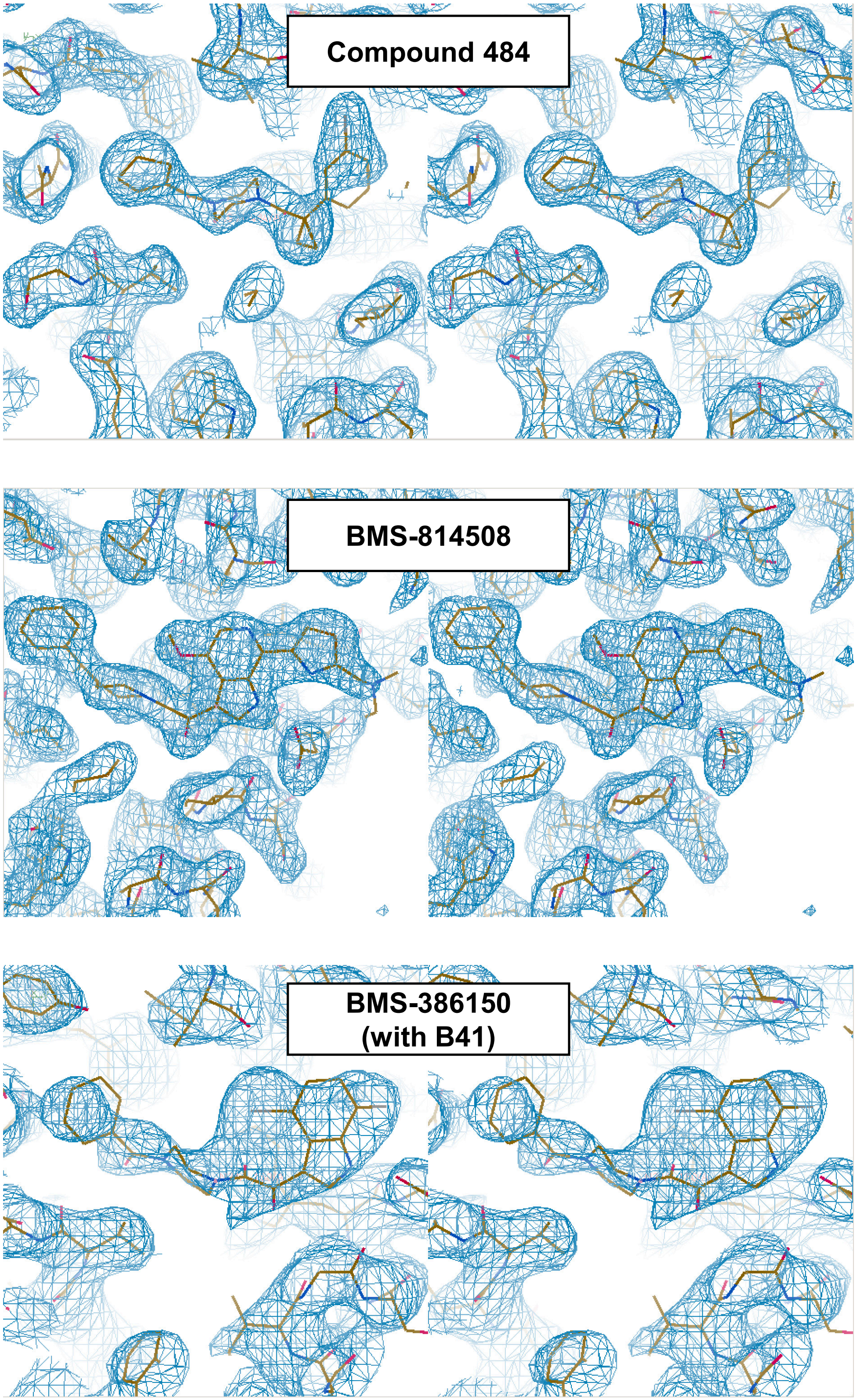

Supplementary Figure 13 (Continued) | Stereo images of the entry inhibitor binding site for various drug binding. 2Fo-Fc maps, contoured at 1.5σ, are shown.

Virus ID	Clade	BMS-818251	BMS-626529
0260.v5.c36	A	0.0010	0.0290
0330.v4.c3	A	0.0100	0.0980
0439.v5.c1	A	0.0006	0.0090
3365.v2.c20	A	0.0900	1.9200
3415.v1.c1	A	0.0004	0.0050
3718.v3.c11	A	0.0007	0.0100
398-F1.F6.20	A	0.0110	0.3720
BB201.B42	A	0.0040	0.2420
BB539.2B13	A	0.0002	0.0070
BG505.W6M.C2	A	0.0003	0.0090
BI369.9A	A	0.0003	0.0060
BS208.B1	A	0.0003	0.0170
KER2008.12	A	0.0150	1.8700
KER2018.11	A	0.0003	0.0080
KNH1209.18	A	0.0004	0.0290
MB201.A1	A	0.0090	0.6280
MB539.2B7	A	0.0020	0.1910
MI369.A5	A	0.0003	0.0130
MS208.A1	A	0.0003	0.0110
Q23.17	A	0.0005	0.0120
Q259.17	A	0.0070	1.2300
Q769.d22	A	0.0010	0.1230
Q769.h5	A	0.0020	0.4270
Q842.d12	A	0.0004	0.0170
QH209.14M.A2	A	0.0600	0.6100
RW020.2	A	0.0007	0.0710
UG037.8	A	0.0100	1.0100
246-F3.C10.2	AC	0.0020	0.2000
3301.v1.c24	AC	0.0050	0.0150
3589.v1.c4	AC	0.0002	0.0010
6540.v4.c1	AC	0.0020	0.0410
6545.v4.c1	AC	0.0005	0.0320
0815.v3.c3	ACD	0.0001	0.0020
6095.v1.c10	ACD	0.0001	0.0010
3468.v1.c12	AD	0.0005	0.0070
Q168.a2	AD	0.0030	0.1150
Q461.e2	AD	0.0003	0.0120
620345.c1	AE	0.0490	4.1300
BJOX009000.02.4	AE	0.1050	5.1300
BJOX010000.06.2	AE	1.6600	>20
BJOX025000.01.1	AE	0.1780	>20
BJOX028000.10.3	AE	0.2360	>20
C1080.c3	AE	5.3800	>20
C2101.c1	AE	1.4700	>20
C3347.c11	AE	0.4520	18.1000
C4118.09	AE	0.0590	4.0400
CM244.ec1	AE	0.2600	>20
CNE3	AE	0.0610	8.5200
CNE5	AE	0.4650	12.7000
CNE55	AE	0.2820	4.7700
CNE56	AE	0.7970	>20
CNE59	AE	0.5060	>20
CNE8	AE	2.6800	>20
M02138	AE	0.0280	2.8100
R1166.c1	AE	>20	>20
R2184.c4	AE	2.0400	>20
R3265.c6	AE	0.1220	13.0000
TH023.6	AE	>20	>20
TH966.8	AE	0.9230	>20
TH976.17	AE	1.2800	>20
235-47	AG	0.0001	0.0010
242-14	AG	0.0005	0.0230
263-8	AG	0.0001	0.0020
269-12	AG	0.6620	15.1000
271-11	AG	1.2400	>20
928-28	AG	0.0460	2.0800
DJ263.8	AG	0.0002	0.0110
T250-4	AG	0.0004	0.0150
T251-18	AG	0.0006	0.0190
T253-11	AG	0.0001	0.0010
T255-34	AG	0.0020	0.0820
T257-31	AG	0.0010	0.0570
T266-60	AG	0.0420	1.3700
T278-50	AG	0.0001	0.0002
T280-5	AG	0.0004	0.0100
T33-7	AG	0.2520	2.8100

Virus ID	Clade	BMS-818251	BMS-626529
3988.25	B	0.0001	0.0010
5768.04	B	0.0003	0.0030
6101.10	B	0.0001	0.0010
6535.3	B	0.0000	0.0003
7165.18	B	0.0110	0.0290
45_01dG5	B	0.0002	0.0004
89.6.DG	B	0.0001	0.0020
AC10.29	B	0.0002	0.0030
ADA.DG	B	0.0001	0.0030
Bal.01	B	*0.0000*	0.0010
Bal.26	B	0.0001	0.0008
BG1168.01	B	0.0010	0.0130
BL01.DG	B	2.9600	>20
BR07.DG	B	0.6410	0.7860
BX08.16	B	0.0001	0.0020
CAAN.A2	B	0.0001	0.0010
CNE10	B	0.0001	0.0005
CNE12	B	0.0020	0.0010
CNE14	B	1.1200	0.2300
CNE4	B	4.7300	>20
CNE57	B	0.0003	0.0020
HO86.8	B	0.0040	0.0200
HT593.1	B	0.0001	0.0009
HXB2.DG	B	0.0001	0.0010
JRCSF.JB	B	0.0000	0.0003
JRFL.JB	B	0.0001	0.0004
MN.3	B	0.0006	0.0100
PVO.04	B	0.0004	0.0030
QH0515.01	B	0.0310	0.0120
QH0692.42	B	0.0007	0.0090
REJO.67	B	0.5900	1.0400
RHPA.7	B	0.0005	0.0040
SC422.8	B	0.0002	0.0020
SF162.LS	B	0.0001	0.0010
SS1196.01	B	0.5790	7.0500
THRO.18	B	0.0002	0.0030
TRJO.58	B	0.0001	0.0010
TRO.11	B	0.1270	1.0800
WITO.33	B	0.0008	0.0050
X2278.C2.B6	B	0.4400	1.2600
YU2.DG	B	0.0005	0.0009
BJOX002000.03.2	BC	0.0002	0.0050
CH038.12	BC	0.0920	0.3290
CH070.1	BC	0.0008	0.0230
CH117.4	BC	0.0004	0.0260
CH119.10	BC	0.0001	0.0040
CH181.12	BC	0.0001	0.0010
CNE15	BC	0.0001	0.0009
CNE19	BC	0.0001	0.0020
CNE20	BC	0.0060	0.3390
CNE21	BC	0.0003	0.0020
CNE40	BC	0.0010	0.0380
CNE7	BC	0.0004	0.0130
286.36	C	0.0001	0.0010
288.38	C	0.0002	0.0030
0013095-2.11	C	0.0001	0.0050
001428-2.42	C	0.0140	0.0490
0077.v1.c16	C	0.0002	0.0020
00836-2.5	C	12.2000	>20
0921.v2.c14	C	0.0001	0.0009
16055-2.3	C	0.0003	0.0030
16845-2.22	C	0.0001	0.0010
16936-2.21	C	0.0004	0.0060
25710-2.43	C	0.0001	0.0020
25711-2.4	C	0.0010	0.0130
25925-2.22	C	0.0001	0.0020
26191-2.48	C	0.0001	0.0020
3168.v4.c10	C	0.0001	0.0010
3637.v5.c3	C	0.0002	0.0010
3873.v1.c24	C	0.0001	0.0006
426c	C	0.0006	0.0300
6322.v4.c1	C	0.0420	0.0910
6471.v1.c16	C	0.0001	0.0020
6631.v3.c10	C	0.0001	0.0030
6644.v2.c33	C	0.0020	0.0710
6785.v5.c14	C	0.0060	0.2920

Virus ID	Clade	BMS-818251	BMS-626529
6838.v1.c35	C	0.0004	0.0190
96ZM651.02	C	0.0001	0.0040
BR025.9	C	0.8800	>20
CAP210.E8	C	0.0140	0.0770
CAP244.D3	C	0.0001	0.0070
CAP256.206.C9	C	0.0001	0.0020
CAP45.G3	C	0.0040	0.0320
Ce1176.A3	C	0.0009	0.0100
CE703010217.B6	C	0.0001	0.0020
CNE30	C	0.0002	0.0010
CNE31	C	0.0010	0.0230
CNE53	C	0.0000	0.0005
CNE58	C	0.0002	0.0040
DU123.06	C	0.0003	0.0050
DU151.02	C	0.0002	0.0060
DU156.12	C	0.0002	0.0030
DU172.17	C	0.0001	0.0080
DU422.01	C	0.0007	0.0080
MW965.26	C	0.0060	0.4330
SO18.18	C	0.0002	0.0030
TV1.29	C	0.0010	0.0670
TZA125.17	C	0.0010	0.0140
TZBD.02	C	0.0003	0.0050
ZA012.29	C	0.0005	0.0070
ZM106.9	C	0.0020	0.0170
ZM109.4	C	0.0001	0.0010
ZM135.10a	C	0.1530	0.8890
ZM176.66	C	0.0010	0.0180
ZM197.7	C	0.0002	0.0060
ZM214.15	C	0.0002	0.0020
ZM215.8	C	0.0001	0.0020
ZM233.6	C	0.0001	0.0008
ZM249.1	C	0.0004	0.0180
ZM53.12	C	0.0004	0.0100
ZM55.28a	C	0.0003	0.0060
3326.v4.c3	CD	0.0001	0.0003
3337.v2.c6	CD	0.0002	0.0010
3817.v2.c59	CD	0.0080	0.0480
191821.E6.1	D	0.0001	0.0008
231965.c01	D	0.0001	0.0010
247-23	D	0.0005	0.0110
3016.v5.c45	D	*0.0000*	0.0005
57128.vrc15	D	0.0006	0.0060
6405.v4.c34	D	0.2570	2.4700
A03349M1.vrc4a	D	0.0001	0.0040
A07412M1.vrc12	D	0.0010	0.0080
NKU3006.ec1	D	0.0002	0.0050
UG021.16	D	0.0002	0.0130
UG024.2	D	>20	0.5660
P0402.c2.11	G	0.0002	0.0320
P1981.C5.3	G	0.0005	0.2090
X1193.c1	G	0.0006	0.1200
X1254.c3	G	0.0002	0.0430
X1632.S2.B10	G	0.0003	0.0540
X2088.c9	G	0.0003	0.0030
X2131.C1.B5	G	0.0010	0.2670
SIVmac251.30.SG3	NA	>20	>20
SVA.MLV	NA	>20	>20

	BMS-818251	BMS-626529
**# Viruses**	208	208
**Total Neutralized**		
**IC50 <50ug/ml**	205	189
**IC50 <10ug/ml**	204	185
**IC50 <1.0ug/ml**	194	167
**IC50 <0.1ug/ml**	173	147
**IC50 <0.01ug/ml**	155	95
**% Neutralized**		
**IC50 <50ug/ml**	99	91
**IC50 <10ug/ml**	98	89
**IC50 <1.0ug/ml**	93	80
**IC50 <0.1ug/ml**	83	71
**IC50 <0.01ug/ml**	75	46
**Median IC50**	0.0005	0.0090
**Geometric Mean**	0.0015	0.0159

IC50 uM
<0.001
.001-.01
.01-.100
.100-1.00
1.00-10.0
>10.0

Supplementary Table 1 | Neutralization of a 208-virus panel by BMS-818251, in comparison to BMS-626529 (*Nature Chemical Biology* (2017) 13(10):1115).

Lattice	Publication	PDB code	Antibodies used to aid crystallization	Lattice contact	Resolution (best direction reported in PDB)	Effective resolution (un-truncated value in parenthesis)
C2	Julien (2013)	4NCO	PGT122*	Ab-Ab (x3)<br>Trimer-Trimer	4.7 Å	4.9 Å
$P6_3$	Pancera (2014)	4TVP	35O22 &<br>PGT122*	Ab-Ab(x3)<br>Ab-Trimer (x1; non-native)	3.1 Å	3.8 Å (3.4Å)
	Garces (2015)	5CEZ	35O22 &<br>3H109L*	Ab-Ab(x3)<br>Ab-Trimer (x1; non-native)	3.0 Å	(3.1 Å)
	Stewart-Jones (2016)	5FYJ-5FYL	35O22 &<br>PGT122*	Ab-Ab(x3)<br>Ab-Trimer (x1; non-native)	3.1 Å	3.3; 3.7; 3.7 Å
$P6_3$	Kwon (2015)	4ZMJ	None	Trimer-Trimer	3.3 Å	3.8 Å
$P2_1$	Scharf (2015)	5CJX	8ANC195	Ab-Trimer (x3; non-native)	3.6 Å (isotropic)	3.6 Å
R32	Xu (2016)	5I8H	PGT122* &<br>VRC34	Ab-Ab (x3)<br>Trimer-Trimer	4.3 Å	5.3 Å
H3	Gristick (2016)	5T3X, 5T3Z	IOMA &<br>10-1074*	Ab-Ab<br>Ab-Trimer	3.5 Å (isotropic)<br>3.9 Å (isotropic)	3.5 Å<br>3.9 Å

* PGT121 family members

Supplementary Table 2 | Published crystal lattices of HIV-1 Env trimer.

	4TVP	5FYL	5CEZ	New
Publication	Pancera *et al*<br>(Kwong lab)<br>2014 *Nature*	Stewart-Jones *et al*<br>(Kwong lab)<br>2016 *Cell*	Garces *et al*<br>(Wilson lab)<br>2015 *Immunity*	Current study
Unit cell dimension<br>(Unit cell volume)	129, 129, 313<br>(34,968)	130, 130, 313<br>(35,239)	128, 128, 316<br>(35,029)	131, 131, 317<br>(35,963)
Resolution by Anisotropy server	4.2, 4.2, 3.1	4.3, 4.3, 2.9	3.3, 3.5, 2.9	3.1, 3.1, 2.3
# of reflections<br>(after truncation)	29,370	28,004	47,098	51,309
# of atoms in the model	12,188	12,718	12,135	10,292
**Data-to-parameter ratio**	**29,370/12,188 =<br>2.41**	**28,004/12,718 =<br>2.20**	**47,098/12,135 =<br>3.88**	**51,309/10,292 =<br>4.99**

Supplementary Table 3 | Ratio between observed unique diffraction and the refined parameters. The new lattice is compared with three other BG505 SOSIP.664 structures (PDB codes: 4TVP, 5FYL and 5CEZ) determined in related lattice.

Supplementary Table 4 | Data collection and refinement statistics (molecular replacement). (Continued on next two pages).

	**BMS-378806** in complex with BG505+35O22_3T2S+3H109L_MM	**Compound 484** in complex with BG505+35O22_3T2S+3H109L_MM
**PDB id**	6MTJ	6MTN
**Data collection**		
Space group	$P6_3$	$P6_3$
Cell dimensions		
a, *b*, *c* (Å)	132.3, 132.3, 315.6	131.5, 131.5, 35.7
α, β, γ (°)	90, 90, 120	90, 90, 120
Resolution (Å)	50.0-2.90 (2.95-2.90) *#; *50.0-2.33 (2.37-2.33)* &	50.0-3.00 (3.05-3.00); *50.0-2.50 (2.54-2.50)* &
R_{sym} or R_{merge} (%)	14.1 (106.7); *13.9 (66.5)*	13.5 (143.0); *13.0 (114.0)*
R_{pim} (%)	6.9 (58.1); *6.8 (58.5)*	7.2 (85.7); *6.9 (74.2)*
$I / \sigma I$	7.7 (2.1); *7.7 (0.8)*	7.4 (2.0); *7.8 (0.6)*
$CC_{1/2}$ (%)	98.5 (84.9); *98.3 (49.1)*	96.7 (65.4); *98.3 (36.2)*
Completeness (%)	97.6 (55.3); *54.0 (1.5)*	95.0 (49.4); *50.9 (2.2)*
Redundancy	5.1 (3.7); *5.0 (1.7)*	4.3 (3.0); *4.4 (3.0)*
Effective resolution ψ	2.92; *2.86*	3.05; *3.13*
Measured reflections	341,519; *355,894*	250,634; *241,947*
Unique reflections	67,511; *71,849*	58,850; *54,831*
**Refinement**		
Resolution (Å)	43.27 - 2.34	43.04 - 2.50
No. reflections	51,309	41,555
R_{work} / R_{free} (%)	23.6 / 28.3	22.3 / 26.3
No. atoms	10,263	10,143
Protein	9,639	9,597
Ligand/ion	548	543
Water	76	3
B-factors (Å^2)	39	43
Protein	38	42
Ligand/ion	56	61
Water	23	31
R.m.s. deviations		
Bond lengths (Å)	0.003	0.004
Bond angles (°)	0.676	0.770

* Data processing statistics based on the overall resolution cutoff determined as: completeness greater than 50% and I/σI greater than 2.

\# Statistics for the highest-resolution shell are shown in parentheses.

& Statistics in italic font were calculated after elliptical truncation by the UCLA anisotropy server.

ψ Effective resolution was calculated by Res_{eff} = (Highest resolution) × (completeness)$^{-1/3}$

Supplementary Table 4 | Data collection and refinement statistics (molecular replacement). (Continued on next page).

	**BMS-814508** in complex with BG505+35O22_3T2S+3H109L_MM	**BMS-818251** in complex with BG505+35O22_3T2S+3H109L_MM	**BMS-386150** in complex with BG505+35O22_3T2S+3H109L_MM
**PDB id**	6MU6	6MU7	6MU8
**Data collection**			
Space group	$P6_3$	$P6_3$	$P6_3$
Cell dimensions			
a, *b*, *c* (Å)	131.3, 131.3, 314.1	131.5, 131.5, 314.3	131.4, 131.4, 315.5
α, β, γ (°)	90.0, 90.0, 120.0	90.0, 90.0, 120.0	90.0, 90.0, 120.0
Resolution (Å)	50-3.2 (3.26-3.20) *#; *50.0-2.55 (2.59-2.55)* &	50-3.1 (3.15-3.10) *#; *50.0-2.50 (2.54-2.50)* &	50-3.5 (3.56-3.50) *#; *50.0-2.99 (3.04-2.99)* &
R_{sym} or R_{merge}	7.1 (66.9); *7.4 (82.4)*	9.0 (114.7); *9.3 (65.9)*	8.3 (161.4); *8.3 (44.3)*
R_{pim}	3.7 (37.2); *3.8 (70.7)*	5.6 (80.4); *5.9 (62.8)*	4.9 (104.7); *4.9 (44.3)*
$I / \sigma I$	17.1 (2.0); *17.4 (1.2)*	9.8 (1.9); *8.9 (0.9)*	12.5 (2.0); *12.4 (1.9)*
$CC_{1/2}$ (%)	99.9 (90.7); *98.8 (55.5)*	98.2 (80.7); *97.8 (48.6)*	98.9 (59.1); *97.8 (35.9)*
Completeness (%)	92.8 (56.8); *50.9 (1.7)*	90.7 (51.7); *55.1 (3.1)*	88.7 (52.3); *58.1 (2.5)*
Redundancy	4.5 (3.7); *4.6 (2.1)*	3.1 (2.4); *2.9 (1.3)*	3.6 (2.9); *3.5 (1.1)*
Effective resolution ψ	3.28; *3.19*	3.20; *3.05*	3.64; *3.58*
Measured reflections	215,973; *236,078*	156,740; *170,402*	125,162; *127,618*
Unique reflections	47,656; *51,503*	50,364; *58,228*	34,501; *36,151*
**Refinement**			
Resolution (Å)	42.96 – 2.55	43.05 – 2.50	43.01 – 2.99
No. reflections	38,841	40,596	28,089
R_{work} / R_{free} (%)	23.1 / 26.4	22.3 / 26.1	21.9 / 25.9
No. atoms	10,172	10,225	10,171
Protein	9,616	9,653	9,635
Ligand/ion	548	559	533
Water	8	13	3
B-factors ($Å^2$)	49	37	56
Protein	48	36	55
Ligand/ion	64	52	72
Water	36	22	27
R.m.s. deviations			
Bond lengths (Å)	0.003	0.003	0.003
Bond angles (°)	0.600	0.619	0.634

* Data processing statistics based on the overall resolution cutoff determined as: completeness greater than 50% and I/σI greater than 2.

\# Statistics for the highest-resolution shell are shown in parentheses.

& The diffraction limits determined by the UCLA anisotropy server were shown; merging statistics after the anisotropic truncation is shown in italic font.

ψ Effective resolution was calculated by Res_{eff} = (Highest resolution) × $(\text{completeness})^{-1/3}$

Supplementary Table 4 | Data collection and refinement statistics (molecular replacement).

	**B41**+35O22_3T2S+3H109L_MM	**B41**+35O22_3T2S+3H109L_MM+**BMS-386150**
**PDB id**	6MUF	6MUG
**Data collection**		
Space group	$P6_3$	$P6_3$
Cell dimensions		
a, *b*, *c* (Å)	132.0, 132.0, 314.7	132.8, 132.8, 313.4
α, β, γ (°)		
Resolution (Å)	50-3.4 (3.46-3.40) *#; *50-2.90 (2.95-2.90)* &	50-3.8 (3.87-3.80) *#; *50-2.93 (2.98-2.93)* &
R_{sym} or R_{merge}	8.0 (68.5); *7.6 (107.9)*	13.5 (44.7); *15.4 (123.0)*
R_{pim}	4.3 (35.0); *4.1 (48.3)*	8.9 (28.5); *10.0 (73.5)*
$I / \sigma I$	10.7 (1.9); *11.8 (1.6)*	6.1 (2.0); *6.1 (0.8)*
$CC_{1/2}$ (%)	99.2 (87.2); *100 (53.7)*	96.9 (75.5); *98.9 (35.8)*
Completeness (%)	81.2 (50.3); *43.9 (4.4)*	69.6 (41.8); *36.6 (4.1)*
Redundancy	3.0 (3.3); *3.3 (5.8)*	2.5 (2.7); *2.8 (3.6)*
Effective resolution ψ	3.64; *3.82*	4.29; *4.09*
Measured reflections	102,970; *96,862*	53,631; *68,529*
Unique reflections	34,122; *29,704*	21,717; *24,712*
**Refinement**		
Resolution (Å)	41.84 – 2.90	40.1 – 2.93
No. reflections	26,891	19,711
R_{work} / R_{free} (%)	25.3 / 29.7	23.0 / 29.0
No. atoms	9,931	10,005
Protein	9,486	9,494
Ligand/ion	445	511
Water	n/a	n/a
B-factors (Å^2)	51	57
Protein	49	56
Ligand/ion	80	81
Water	n/a	n/a
R.m.s. deviations		
Bond lengths (Å)	0.003	0.002
Bond angles (°)	0.687	0.529

* Data processing statistics based on the overall resolution cutoff determined as: completeness greater than 50% and I/σI greater than 2.

\# Statistics for the highest-resolution shell are shown in parentheses.

& The diffraction limits determined by the UCLA anisotropy server were shown; merging statistics after the anisotropic truncation is shown in italic font.

ψ Effective resolution was calculated by Res_{eff} = (Highest resolution) × (completeness)$^{-1/3}$
